# Safety and nonclinical and clinical pharmacokinetics of PC945, a novel inhaled triazole antifungal agent

**DOI:** 10.1002/prp2.690

**Published:** 2020-12-19

**Authors:** Lindsey Cass, Alison Murray, Amanda Davis, Kathy Woodward, Muna Albayaty, Kazuhiro Ito, Pete Strong, John Ayrton, Charlie Brindley, Jayne Prosser, John Murray, Eddie French, Phillip Haywood, Christopher Wallis, Garth Rapeport

**Affiliations:** ^1^ Pulmocide Ltd London UK; ^2^ Parexel Early Phase Clinical Unit Level 7 Northwick Park Hospital Harrow UK; ^3^ KinetAssist Limited Quothquan UK

**Keywords:** antifungal, drug‐drug interaction, first‐in‐human, inhaled administration, PC945, pharmacokinetics, safety

## Abstract

PC945 is a novel antifungal triazole formulated for nebulized delivery to treat lung *Aspergillus* infections. Pharmacokinetic and safety profiles from nonclinical studies and clinical trials in healthy subjects, and subjects with mild asthma were characterized. Toxicokinetics were assessed following daily 2‐hour inhalation for 14 days. Potential for drug‐drug interactions was evaluated using pooled human liver microsomes. Clinical safety and pharmacokinetics were assessed following (a) single inhaled doses (0.5‐10 mg), (b) 7‐day repeat doses (5 mg daily) in healthy subjects; (c) a single dose (5 mg) in subjects with mild asthma. *C*_max_ occurred 4 hours (rats) or immediately (dogs) after a single dose. PC945 lung concentrations were substantially higher (>2000‐fold) than those in plasma. PC945 only inhibited CYP3A4/5 substrate metabolism (IC_50_: 1.33 µM [testosterone] and 0.085 µM [midazolam]). Geometric mean *C*_max_ was 322 pg/mL (healthy subjects) and 335 pg/mL (subjects with mild asthma) 4‐5 hours (median t_max_) after a single inhalation (5 mg). Following repeat, once daily inhalation (5 mg), Day 7 *C*_max_ was 951 pg/mL (0.0016 µM) 45 minutes after dosing. Increases in *C*_max_ and AUC_0–24h_ were approximately dose‐proportional (0.5‐10 mg). PC945 administration was well tolerated in both healthy subjects and subjects with mild asthma. Treatment‐emergent adverse events were mild/moderate and resolved before the study ended. No clinically significant lung function changes were observed. PC945 pharmacokinetics translated from nonclinical species to humans showed slow absorption from lungs and low systemic exposure, thereby limiting the potential for adverse side effects and drug interactions commonly seen with systemically delivered azoles.

## Graphical abstract

PC945 pharmacokinetics translated from nonclinical species to humans showed slow absorption from lungs and low systemic exposure, thereby limiting the potential for adverse side effects and drug‐drug interactions commonly seen with systemically delivered azole antifungals.

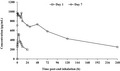

**Abbreviations**
AUC_0–24h_: area under the plasma concentration versus time curve from 0 to 24 hours postdose
*C*_max_: maximum observed concentration
CYP450: cytochrome P450
FEV_1_: forced expiratory volume over 1 second
FVC: forced vital capacity
R_o_: accumulation ratio
*t*_½_: apparent terminal half‐life
t_max_: time to *C*_max_

**What is already known about this subject**

- The high plasma drug concentrations required to treat pulmonary fungal infections with current systemic antifungal therapies are commonly associated with side effects and drug‐drug interactions.
- PC945, a novel triazole, inhibits planktonic growth and bronchial epithelial cell infection by *Aspergillus* species in vitro.

**What this study adds**

- Findings from clinical and nonclinical studies demonstrated that repeat daily doses of inhaled PC945 led to prolonged absorption from the lung and minimal systemic exposure.
- PC945 was well tolerated in healthy subjects and subjects with mild asthma.
- Inhaled PC945 should have a wider therapeutic index than systemic antifungal agents.

## 1 INTRODUCTION

Incidence of fungal infections has increased substantially over the past two decades.^1^ Immunocompromised or immunosuppressed patients are particularly susceptible to such infections, and invasive forms remain a leading cause of morbidity and mortality for these patients.^2^ Pulmonary aspergillosis, caused by *Aspergillus*, is particularly problematic.^3^ *Aspergillus fumigatus* is one of the primary causative agents of lung infections in humans.^4^ Chronic *Aspergillus* infections can leave patients with permanent lung damage, requiring life‐long management using oral azole treatment.^5^

Existing treatments for fungal infections are administered orally (azoles) or intravenously (azoles, amphotericin B or the echinocandins).^6^ Azole antifungals are potent inhibitors of cytochrome P450 (CYP450) enzymes, especially the CYP3A4 isoenzyme, which is responsible for the metabolism of a broad range of drugs.^7^ This inhibition presents a significant risk of interactions with other comedicated drugs. To allow orally or intravenously administered antifungal agents to achieve high local concentrations sufficient for pathogen clearance, systemic exposure must be high, resulting in poor safety profiles.^8^, ^9^

Nebulized delivery of antifungal agents results in higher local exposure in the epithelial lining fluid compared with intravenous administration.^10^ PC945 is the first antifungal triazole specifically designed to treat pulmonary infection via inhaled administration.^11^ In common with other triazole agents, PC945 inhibits the enzyme lanosterol 14α‐demethylase (CYP51A1) in fungus, which prevents conversion of lanosterol to ergosterol.^12^, ^13^ Reduction of ergosterol causes disruption to the structure and function of fungal membranes, hence inhibiting fungal growth and spread. Using a method from the European Committee on Antimicrobial Susceptibility Testing (EUCAST), against 96 clinically isolated *A. fumigatus* strains obtained in France and the United Kingdom, geometric mean minimum inhibitory concentration (MIC) of PC945 was 0.17 μg/mL, and MIC_50_ and MIC_90_ values were 0.125 and 1.0 μg/mL, respectively; the potency of PC945 was superior to that of voriconazole and comparable to that of posaconazole.^13^ PC945 was also found to inhibit *A. fumigatus* infection in an in vitro human alveolus bilayer model.^14^ In animal models, PC945 delivered a sustained and persistent antifungal effect in the lung when administered intranasally.^15^ In addition, PC945 was more effective than alternative antifungal agents because of a higher local exposure at the infected site, which was attributed to the intranasal administration delivering the compound directly to the lung.^13^, ^15^

In this article, we report the pharmacokinetic profile of PC945 in nonclinical studies after single and repeat inhaled doses in rats and dogs. We also present the results from a Phase 1 study, which evaluated the safety, tolerability and pharmacokinetics of single (escalating), and repeat inhaled doses of PC945 in healthy subjects, as well as the safety and tolerability of a single dose of inhaled PC945 in subjects with mild asthma (ClinicalTrials.gov Identifier: NCT02715570). It was important to demonstrate a low systemic exposure (ie, in pg/mL level) following inhaled delivery to ensure a favorable safety profile for PC945 compared with the current systemic (oral and intravenous) treatments.

## 2 MATERIALS AND METHODS

### 2.1 Drug

For nonclinical studies, PC945 was synthesized by Sygnature Discovery Ltd (Nottingham, UK) and micronized at JetPharma (Balerna, Switzerland). PC945 powder was directly suspended in sodium phosphate‐buffered saline containing wetting agents to 10 mg/mL and further diluted with physiological saline after sonication to obtain the desired dose concentration.

For the clinical study, PC945 was manufactured by Onyx Scientific Ltd (Sunderland, UK), micronized at JetPharma and supplied as a powder at a single strength of 14 mg/vial (Juniper Pharma, Nottingham, UK) for reconstitution using placebo solution. Placebo was supplied in a similar vial (Nova Laboratories, Leicester, UK). PC945 and placebo were administered by oral inhalation using a PARI LC SPRINT^®^ nebuliser and PARI TurboBoy SX^®^ compressor (PARI Medical Ltd., Surrey, UK). The particle size distribution of the drug substance in the product has been designed to be typical of inhaled medicines that are used to treat lung disease and is expected to be able to penetrate small airways, ie, a d50 of <2 µm and a d90 of <4.5 µm when measured by laser diffraction.

### 2.2 Nonclinical toxicokinetic studies

All animal studies were designed to meet the requirements of European Parliament and Council Directive 2001/83/EC and its amendment, Commission Directive 2003/63/EC,^16^, ^17^ and were conducted in accordance with the applicable sections of the United Kingdom Animals (Scientific Procedures) Act 1986, Amendment Regulations 2012 (the Act).

PC945 was administered to rats (Han Wistar) and dogs (Beagle) at nominal doses of approximately 2, 7, and 18 mg/kg. Each dose was inhaled over a 2‐hour period once daily for 14 days. Blood samples (0.3 mL for rats and 0.5 mL for dogs) were collected at six timepoints ranging from 0 to 22 hours postend of inhalation on Day 1 (single dose systemic exposure) and on Day 14 (repeat dose exposure). Lung samples were taken from rats on Day 15 (~22 hours after the end of the final dose) to measure PC945 lung concentrations.

### 2.3 In vitro drug‐drug interactions

PC945 and posaconazole (Sigma‐Aldrich, UK) were added to the pooled human liver microsomal samples (Pharmaron Rushden, UK) at concentrations of 0, 0.03, 0.1, 0.3, 1, 3, and 5 μmol/L. The mixtures were preincubated at 37°C for 0 or 30 minutes before the addition of selective CYP substrates. Microsomal reactions were terminated by adding 100 μL of cold (4°C) methanol containing appropriate internal standard. Aliquots (150 μL) of each sample were centrifuged and the supernatant was used for substrate analysis by ultra‐performance liquid chromatography with tandem mass spectrometry (UPLC‐MS/MS) on the day of incubations.

Change in substrate concentration was measured to determine the extent of CYP‐isoform inhibition. The 30‐minute preincubations were performed to determine whether there was time‐dependent inhibition of any CYP isoform (CYP1A2, 2B6, 2C8, 2C9, 2C19, 2D6, and 3A4/5). Untreated control contained only solvent, dimethyl sulfoxide (≤0.5% [v/v]). Positive control contained a chemical inhibitor selective for each CYP enzyme in the presence of PC945 or posaconazole.

### 2.4 In vitro plasma protein binding

Plasma protein binding of PC945 was determined by ultrafiltration in pooled plasma samples from human, dog, rat, and mouse. PC945 was tested at 0.1, 1, 5, 25, and 50 µmol/L in plasma for each species. PC945‐containing plasma samples were centrifuged at 238 859 *g* for 20 hours at 37°C. Supernatant was removed and diluted. The concentration of unbound PC945 was determined using LC‐MS/MS, from which the extent of protein binding was calculated.

### 2.5 Clinical trial

#### 2.5.1 Trial design

This PC945 first‐in‐human clinical trial (ClinicalTrials.gov Identifier: NCT02715570) was a two‐part randomized, placebo‐controlled study to assess the safety and tolerability of PC945 in both healthy subjects and subjects with mild asthma, conducted at Parexel Early Phase Clinical Unit (Harrow, UK). The study was considered single‐blind as the appearance of active and placebo doses were different, however, it was conducted in a double‐blind manner (employing separate dosing and assessment teams).

Part one consisted of a single dose escalation study (Cohort 1) and a repeat dose study (Cohort 2) of inhaled PC945 in healthy subjects. Part two comprised a single dose study of inhaled PC945 in subjects with mild asthma (Cohort 3). The doses selected were predicted to achieve target lung concentrations above the IC_90_/MIC_90_ for *A. fumigatus* (based on data from a range of in vitro and in vivo systems) throughout a 24‐hour period and with a suitable safety margin based on animal toxicology data.

The study was carried out in accordance with the Declaration of Helsinki, the International Council for Harmonisation consolidated guideline for Good Clinical Practice, and local regulations and were approved by an Independent Ethics Committee. All subjects signed a written informed consent form before enrollment.

**FIGURE 1:**
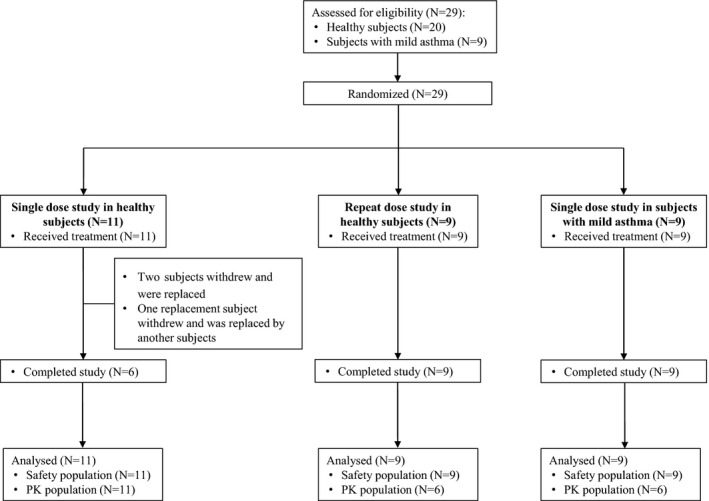
Subject disposition (CONSORT diagram)

In Cohort 1, 11 healthy subjects (three males and eight females; mean age: 48.5 years, mean weight: 65.32 kg, mean body mass index [BMI]: 23.62 kg/m^2^), attended the single ascending dose study. Eight healthy subjects started the study in which two subjects were randomized to each of the four ascending dose treatment sequences (Table 1); two subjects withdrew due to adverse events unrelated to study medication and both were replaced. One of the replacement subjects subsequently withdrew from the study for personal reasons and was replaced by another subject (Figure 1). Overall, six subjects each received three of four doses of PC945 (0.5, 2, 5, and 10 mg) and one dose of placebo. These doses were administered with a minimum of 13 days interval to allow for washout.

**TABLE 1:** Treatment sequences for the single ascending dose study (Cohort 1)

Week 1^a^, ^b^	Week 2^a^, ^b^	Week 3^a^, ^b^	Week 4^a^, ^b^
PC945 0.5 mg	PC945 2 mg	PC945 5 mg	Placebo
PC945 0.5 mg	PC945 2 mg	Placebo	PC945 10 mg
PC945 0.5 mg	Placebo	PC945 5 mg	PC945 10 mg
Placebo	PC945 2 mg	PC945 5 mg	PC945 10 mg

^a^ Sentinel dosing: two subjects (PC945:placebo = 1:1) were dosed on Day 1; the remaining six subjects were dosed on Day 2 for each week of treatment.

^b^ PC945 was supplied as a powder for reconstitution (using placebo solution) at a single strength of 14 mg/vial to achieve the target doses. A single 14 mg vial when reconstituted to a concentration of 4 mg/mL delivers 5 mg of PC945 to the patient from the nebulizer as determined using an in vitro breath simulator.

Nine healthy subjects (four males and five females; mean age: 33.2 years, mean weight: 66.5 kg, mean BMI: 23.53 kg/m^2^) whose forced expiratory volume over 1 second (FEV_1_) and forced vital capacity (FVC) were ≥80% of predicted values with an FEV_1_/FVC ratio >0.7, received once daily doses of PC945 5 mg or placebo for 7 days (Cohort 2, PC945:placebo = 2:1).

In Cohort 3, nine subjects with mild asthma (six males and three females; mean age: 37.7 years, mean weight: 82.70 kg, mean BMI: 26.48 kg/m^2^) received a single dose of PC945 5 mg or placebo (PC945:placebo = 2:1). Subjects with mild asthma needed to demonstrate a methacholine PC_20_ (concentration of inhaled agonist leading to a fall in FEV_1_ of ≥20% of personal best) ≤8 mg/mL at screening and an FEV_1_ >60% of predicted normal value at least 6 hours after the last use of a short acting β‐agonist.

Subjects returned to the study unit for a final follow‐up visit 10 days after the last administration of study medication.

#### 2.5.2 Sample collection and handling

Serial blood samples were collected to determine plasma PC945 concentrations. For the single dose study in healthy subjects and in subjects with mild asthma, blood samples were collected predose and at 0.25, 0.5, 1, 2, 4, 6, 8, 24, 30, and 48 hours postend of inhalation and at the follow‐up visit. For the repeat dose study, blood samples were collected (a) predose and at 0.25, 0.5, 1, 2, 4, 6, 8, 10, 12, and 24 hours postend of inhalation on Days 1 and 7, and 30 and 48 hours on Day 7; (b) predose on Days 5 and 6; (c) at the follow‐up visit.

#### 2.5.3 Safety assessment

Safety was evaluated based on assessments of adverse events, physical examination, vital signs, 12‐lead electrocardiogram, spirometry and clinical laboratory tests. The verbatim terms used to identify adverse events were coded using the Medical Dictionary for Regulatory Activities (version 20.1). All adverse events were mapped to system organ class and preferred term.

Spirometry parameters (FEV_1_ and FVC) were measured in all subjects to evaluate the local tolerability and potential for bronchospasm of PC945 in accordance with American Thoracic Society/European Respiratory Society guidelines. Predicted values were calculated using National Health and Nutrition Examination Survey reference equations.^18^, ^19^

### 2.6 Plasma sample analysis

PC945 concentrations were determined by a validated, specific and sensitive LC‐MS/MS method under the supervision of LGC Ltd (Fordham, UK) after the drug was extracted from plasma samples. Lower limit of quantification of the assay was 10 pg/mL for clinical samples and 100 pg/mL for rat and dog plasma samples.

### 2.7 Pharmacokinetic analysis

Plasma pharmacokinetic parameters of PC945 were estimated using a fully validated version of Phoenix WinNonlin^®^ (version 8.0; Certara, NJ, USA). The estimated pharmacokinetic parameters included the maximum observed concentration (*C*_max_), time to *C*_max_ (t_max_), the area under the plasma concentration versus time curve from 0 to 24 hours postdose (AUC_0–24h_), the apparent terminal half‐life (*t*_½_) and the accumulation ratio (R_o_).

Attainment of steady state was assessed by visual inspection of the predose plots during the 7‐day treatment period in human subjects.

### 2.8 Analysis of lung samples

Portions of rat lung tissue were homogenized in methanol:water (50:50; 15 mL for 1 g tissue). PC945 concentrations in tissue homogenates were analyzed using LC‐MS/MS. A control rat lung sample (B&K Universal Ltd, Hull, UK) was used as standards and quality control.

### 2.9 Nomenclature of Targets and ligands

Key protein targets and ligands in this article are hyperlinked to corresponding entries in http://www.guidetopharmacology.org, the common portal for data from the IUPHAR/BPS Guide to PHARMACOLOGY,^20^ and are permanently archived in the Concise Guide to PHARMACOLOGY 2019/20.^21^

## 3 RESULTS

### 3.1 Nonclinical toxicokinetic data

#### 3.1.1 Single dose toxicokinetic studies in rats and dogs

Following inhalation of PC945, the increase in systemic exposure was less than proportional at doses of 2.0, 7.4 and 16.6 mg/kg. Systemic exposure to PC945 was consistently higher in female than in male rats (Table 2). *C*_max_ occurred 6 hours after the start of dosing and PC945 was detectable throughout the 24‐hour sampling period after dosing.

**TABLE 2:** Mean systemic exposure^a^ to PC945 in male and female rats following single inhaled doses

Dose (mg/kg)	Male rats		Female rats	
	*C*_max_ (ng/mL)	AUC_0‐24h_ (ng·h/mL)	*C*_max_ (ng/mL)	AUC_0‐24h_ (ng·h/mL)
2.0	7.3	103	10.5	183
7.1	15.8	217	24.8	416
16.6	26.0	426	41.3	731

Abbreviations: AUC_0‐24h_, area under the plasma concentration curve from time zero to 24 hours postdose; *C*_max_, maximum observed concentration.

^a^ No standard deviations were determined on pharmacokinetic parameters as these were derived from PC945 concentrations in groups of rats per time point.

The magnitude of exposure obtained in dogs was lower than that in rats at similar dose/kg levels. There was a slightly less than dose‐proportional increase in systemic exposure at inhaled PC945 doses of 1.7, 5.3 and 18.1 mg/kg in dogs, and no notable differences in mean values of *C*_max_ and AUC_0‐24h_ between male and female dogs (Table 3). *C*_max_ was reached in the samples taken immediately after the 2‐hour dosing period; exposure extended for the full 24‐hour sampling period at all doses.

**TABLE 3:** Mean (standard deviation) systemic exposure to PC945 in male and female dogs following a single inhaled dose

Dose (mg/kg)	Male dogs		Female dogs	
	*C*_max_ (ng/mL)	AUC_0‐24h_ (ng·h/mL)	*C*_max_ (ng/mL)	AUC_0‐24h_ (ng·h/mL)
1.7	2.2 ± 0.3	17.2 ± 3.5	1.5 ± 0.5	12.2 ± 2.9
5.3	4.6 ± 0.4	38.5 ± 1.8	4.3 ± 0.7	39.0 ± 2.7
18.1	9.7 ± 2.4	88.5 ± 25.8	10.2 ± 1.3	90.6 ± 6.7

Abbreviations: AUC_0‐24h_, area under the plasma concentration curve from time zero to 24 hours postdose; *C*_max_, maximum observed concentration.

#### 3.1.2 Repeat dose toxicokinetic studies in rats and dogs

In both test species, daily systemic exposure was higher on Day 14 than Day 1 following inhaled dosing with PC945 suspension. In rats the R_o_ values for *C*_max_ were similar to those for AUC_0–24h_ (ranging from 1.8‐ to 2.8‐fold) and showed no sex dependence (Table 4). There was a trend towards greater accumulation in the highest dose group tested. In dogs, the R_o_ values for *C*_max_ were lower than those for AUC_0‐24h_ (ranging from 1.3‐ to 2.3‐fold) on Day 14 (Table 5).

**TABLE 4:** Summary of systemic exposure^a^ to PC945 in male and female rats following 14 days of inhaled dosing of 2.0, 7.1 and 16.6 mg/kg/day

Dose (mg/kg)	Male rats			Female rats		
	*C*_max_ (ng/mL)	AUC_0‐24h_ (ng·h/mL)	R_o_ ^b^	*C*_max_ (ng/mL)	AUC_0‐24h_ (ng·h/mL)	R_o_ ^b^
2.0	12.3	202	1.96	19.2	336	1.84
7.1	23.7	424	1.91	50.4	749	1.91
16.6^c^	76.9	1140	2.76	92.3	1850	2.53

Abbreviations: AUC_0‐24h_, area under the plasma concentration curve from time zero to 24 hours postdose; *C*_max_, maximum observed concentration; R_o_, accumulation ratio.

^a^ No standard deviation on pharmacokinetic parameters as these were derived from PC945 concentrations in groups of rats per time point.

^b^ Accumulation ratio (R_o_) derived from AUC_0‐24h_ (Day14)/AUC_0‐24h_ (Day 1). Day 1 data are shown in Table 2.

^c^ The no‐observed‐adverse‐effect level (NOAEL) was the highest dose tested (16.6 mg/kg/day).

**TABLE 5:** Summary of mean^a^ systemic exposure to PC945 in male and female dogs following 14 days of inhaled dosing of 1.7, 5.3 and 18.1 mg/kg/day

Dose (mg/kg)	Male dogs			Female dogs		
	*C*_max_ (ng/mL)	AUC_0‐24h_ (ng·h/mL)	R_o_ ^b^	*C*_max_ (ng/mL)	AUC_0‐24h_ (ng·h/mL)	R_o_ ^b^
1.7	2.8	35.5	2.06	2.3	27.7	2.27
5.3	5.6	70.9	1.84	5.5	73.3	1.88
18.1^c^	13.5	203	2.29	7.8	114	1.26

Abbreviations: AUC_0‐24h_, area under the plasma concentration curve from time zero to 24 hours postdose; *C*_max_, maximum observed concentration; R_o_, accumulation ratio.

^a^ Standard Deviation values were omitted for reasons of clarity in presentation of mean data.

^b^ Accumulation ratio (Ro) was derived from AUC_0‐24h_ (Day14)/AUC_0‐24h_ (Day 1). Day 1 data are shown in Table 3.

^c^ The highest dose tested (18.1 mg/kg/day) was found to be the no‐observed‐effect level (NOEL).

Determination of lung exposure in rats on Day 15 confirmed that the concentration of PC945 in lung samples increased with the daily dose administered. PC945 concentrations in the lung were substantially higher (>2000‐fold) than those detected in plasma at all dose levels. There was no clear sex effect in the magnitude of PC945 concentrations in the Day 14 lung tissue samples; however, the ratio of lung to plasma PC945 concentration was consistently lower in female rats, reflecting the sex effect of apparent clearance for PC945 in rats (Table 6). No systemic toxicity was observed at the highest doses tested; this provides a 12‐fold (rat) and threefold (dog) margin over the clinical AUC following a 5 mg dose.

**TABLE 6:** Summary of mean plasma *C*_max_ and mean lung tissue concentrations of PC945 in male and female rats following 14 days of inhaled dosing of 2.0, 7.1 and 16.6 mg/kg/day

Achieved inhaled dose (mg/kg)	Mean data in male rats			Mean data in female rats		
	Plasma *C*_max_ (ng/mL)	Lung concentration^a^ (ng/mL)	Ratio^b^	Plasma *C*_max_ (ng/mL)	Lung concentration (ng/mL)	Ratio^b^
2.0	12.3	42 400	3450	19.2	50 100	2610
7.1	23.7	119 000	5020	50.4	138 000	2740
16.6	76.9	329 000	4280	92.3	216 000	2340

Abbreviation: *C*_max_, maximum observed concentration.

^a^ Lungs were harvested at approximately 22 hours after the end of the final dose.

^b^ Ratio was derived from lung tissue PC945 concentration (Day 15)/plasma *C*_max_ (Day 14).

#### 3.1.3 In vitro drug‐drug interaction

The only cytochrome P450 inhibitory interaction observed for PC945 was that on CYP3A4/5 substrates. Without preincubation, PC945 showed median inhibitory concentration (IC_50_) values of 1.33 µmol/L and 0.085 µmol/L for testosterone and midazolam, respectively. The control CYP3A4/5 inhibitor posaconazole showed differences to PC945 in both inhibitory potency and specificity, with IC_50_ values of 0.081 and 0.32 µmol/L for testosterone and midazolam, respectively.

Following a 30‐minute preincubation, PC945 demonstrated a fivefold shift in potency for CYP3A4/5 (IC_50_ values: 0.247 µmol/L [ie, 168 ng/mL; testosterone] and 0.017 µmol/L [ie, 11.6 ng/mL; midazolam]). PC945 had no time‐dependent effect on other CYP isoforms.

#### 3.1.4 In vitro plasma protein binding

PC945 was highly bound to plasma proteins in all species and the extent of binding was independent of the concentration over a range of 0.1‐50 µmol/L. Mean extent of protein binding was between 96% and 98% in human, mouse and rat plasma and 91% in dog plasma (Table 7).

**TABLE 7:** Summary of mean and range of cross‐species plasma protein binding

Species	Mean percentage protein bound^a^	Range of % bound values across concentration range
Human	96.6	94.7‐98.6
Dog	91.1	82.3‐97.2
Rat	97.8	96.8‐98.3
Mouse	96.2	93.0‐98.7

^a^ Mean values for protein binding cover the PC945 range of 0.1‐50 µmol/L. No clear trend of concentration dependent binding was observed.

### 3.2 Clinical study data

#### 3.2.1 Safety and tolerability

PC945 was well tolerated following single doses up to 10 mg and repeat doses of 5 mg daily for 7 days in healthy subjects, and at a single dose of 5 mg in subjects with mild asthma. Escalation of single doses of PC945 from 0.5 to 10 mg was not associated with an increase in treatment‐emergent adverse events. All treatment‐emergent adverse events were either mild or moderate in intensity and resolved before the end of the study. There were no deaths or serious adverse events in the study. Treatment‐emergent adverse events attributed to study drug by investigators are summarized in Table 8. In total, seven adverse events required intervention; only one of these was considered drug‐related (headache, following a single 10 mg dose of PC945).

**TABLE 8:** Summary of subjects with treatment‐emergent adverse events considered by investigators to be related to study drug (First‐in‐Human Study)

	Cohort 1 (Single dose) n (%)					Cohort 2 (Once daily for 7 days) n (%)		Cohort 3 (Single dose) n (%)	
	Placebo^a^	PC945	PC945	PC945	PC945	Placebo	PC945	Placebo	PC945
		0.5 mg	2 mg	5 mg	10 mg		5 mg		5 mg
	N = 8	N = 6	N = 6	N = 6	N = 6	N = 3	N = 6	N = 3	N = 6
No. of subjects with treatment‐emergent adverse events attributed to study drug	4 (50.00)	1 (16.67)	0	2 (33.33)	2 (33.33)	1 (33.33)	2 (33.33)	0	0
Diarrhea	0	0	0	0	1 (16.67)	0	0	0	0
Feces soft	1 (12.50)	0	0	1 (16.67)	0	0	0	0	0
Nausea	0	0	0	1 (16.67)	1 (16.67)	0	1 (16.67)	0	0
Fatigue	0	1 (16.67)	0	0	0	0	0	0	0
Muscle spasms	0	0	0	1 (16.67)	0	0	0	0	0
Musculoskeletal discomfort	1 (12.50)	0	0	0	0	0	0	0	0
Dizziness	0	0	0	1 (16.67)	1 (16.67)	0	0	0	0
Headache	1 (12.50)	0	0	1 (16.67)	1 (16.67)	0	1 (16.67)	0	0
Lethargy	0	0	0	1 (16.67)	0	0	0	0	0
Cough	1 (12.50)	0	0	0	0	0	0	0	0
Productive cough	1 (12.50)	0	0	0	0	0	0	0	0
Throat tightness	0	0	0	0	0	1 (33.33)	0	0	0

N = number of subjects per group; n = number of subjects with AEs attributed to study drug.

Only treatment‐emergent adverse events included in this table. All adverse events are coded using medical Dictionary for Regulatory Activities (MedDRA) version 20.1.

^a^ Placebo was pooled within cohort.

No clinically significant changes in lung function were observed in healthy subjects. Importantly, no evidence of acute bronchospasm or significant change in lung function (defined as >15% change from baseline) was observed in any subject with mild asthma who received PC945. One subject had a transient reduction in FEV_1_ values >15% compared with baseline 10 minutes after receiving a dose of placebo.

**FIGURE 2:**
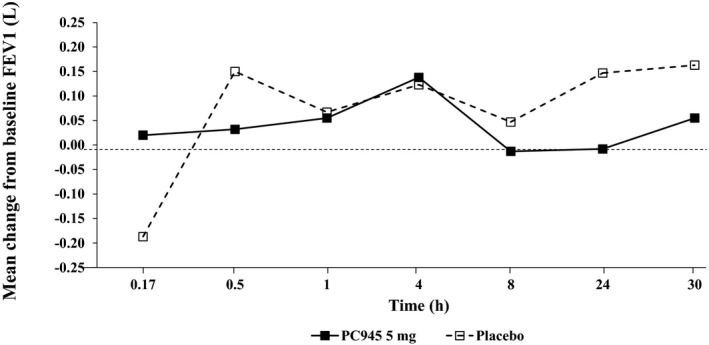
Mean change from baseline in FEV_1_ for subjects with mild asthma (Cohort 3)

There were no notable differences in mean laboratory values, vital signs, electrocardiogram results or spirometry values (Figure 2) between the placebo and PC945 groups. No clinically significant abnormal laboratory results considered to be related to study drug were reported in any cohort.

#### 3.2.2 Pharmacokinetics

##### Single dose in healthy subjects (Cohort 1)

**FIGURE 3:**
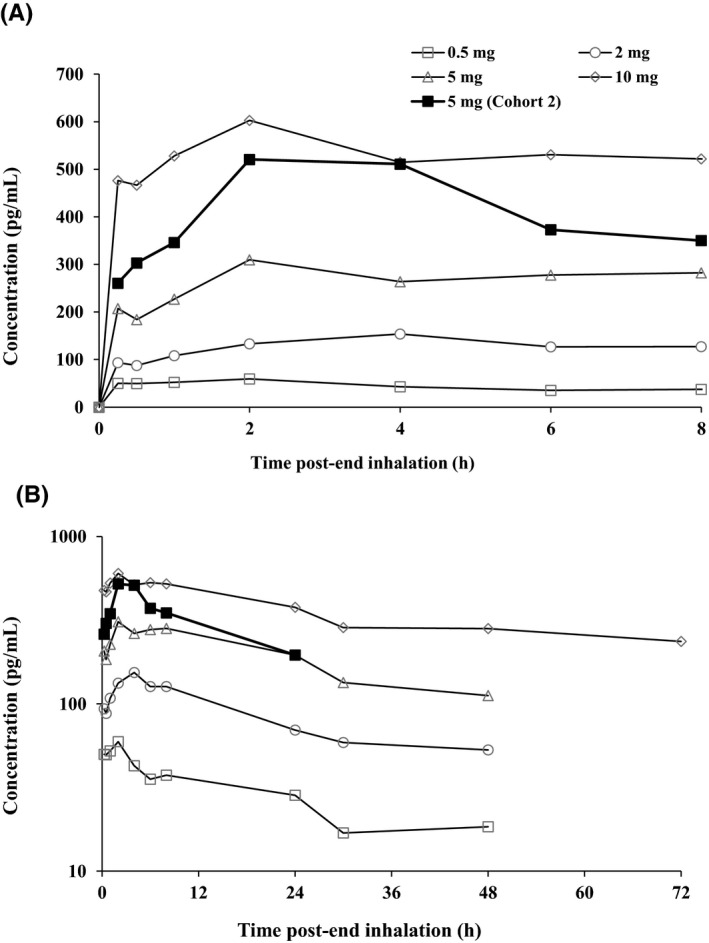
Mean plasma concentrations (linear [A] and log scales [B]) vs time profiles of PC945 after single dose administration in healthy subjects (Cohort 1 and Day 1 of Cohort 2)

Following single inhaled administration of PC945 at 0.5, 2, 5 and 10 mg in healthy subjects, geometric mean *C*_max_ values of 54.7, 128, 322, and 619 pg/mL in plasma were achieved at 1, 4, 5, and 2 hours (median *t*_max_) postend of inhalation, respectively (Table 9 and Figure 3). Geometric mean *t*_½_ ranged from 27.9 to 110 hours over the entire dose range. However, the period over which estimated *t*_½_ was calculated was less than twofold the *t*_½_ itself and the area extrapolated was greater than 20% in most cases; estimated *t*_½_ was therefore considered to be unreliable at each dose level. Between‐subject variability in the extent of systemic exposure to PC945 at 0.5, 2, 5 and 10 mg was moderate to high, as revealed by geometric coefficient of variations for *C*_max_ and AUC_0–24h_ ranging from 39.1% to 107%. Systemic exposure to PC945 in female subjects was not appreciably different to that in male subjects.

**TABLE 9:** Geometric mean (CV%) pharmacokinetic parameters of PC945 following single doses of PC945 in healthy subjects (Cohort 1)

Parameter	0.5 mg (N = 6)	2 mg (N = 6)	5 mg (N = 6)	10 mg (N = 6)
*C*_max_ (pg/mL)	54.7 (64.8)^a^	128 (107)	322 (51.1)	619 (39.1)
*t*_max_ (h)^a^	1.10 (0.250‐2.10)^a^	4.11 (2.09‐8.11)	5.10 (2.08‐8.12)	2.13 (0.330‐6.15)
AUC_0–24h_ (pg•h/mL)	726 (73.4)^a^	1990 (102)	5440 (48.0)	10 600 (40.2)
AUC_0–∞_ (pg•h/mL)	1480 (7.41)^b^	3140 (175)^c^	12 200 (88.0)^d^	58 200 (42.5)
*t*_½_ (h)	36.9 (24.1)^b^	27.9 (58.4)^c^	37.3 (36.4)^d^	110 (16.5)

Note

For t_max_, data are presented as median (range).

Abbreviations: AUC_0–24h_, area under the plasma concentration‐time curve from time zero to 24 hours; AUC_0–∞_, area under the plasma concentration‐time curve from time zero extrapolated to infinity; *C*_max_, maximum plasma concentration; CV, coefficient of variation; *t*_½_, apparent terminal half‐life; t_max_, time at which *C*_max_ occurs.

^a^ One subject had no measurable plasma concentrations of PC945; therefore, was excluded from the concentration data and PK analysis.

^b^ n = 2.

^c^ n = 3.

^d^ n = 4.

##### Single dose in subjects with mild asthma (Cohort 3)

**FIGURE 4:**
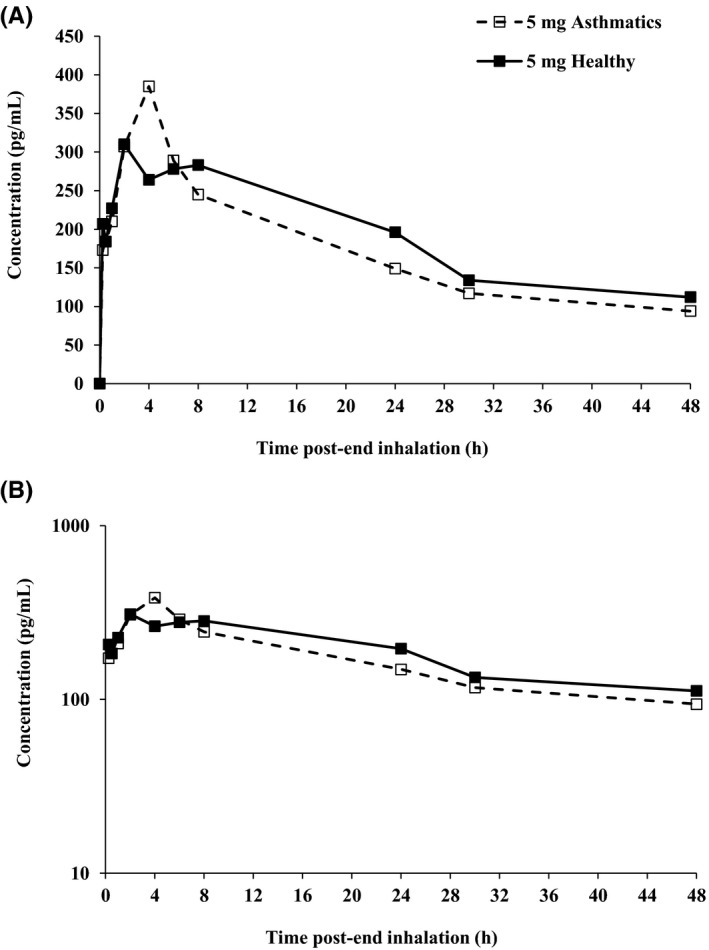
Mean plasma concentrations (linear [A] and log scales [B]) vs time profiles of PC945 following a single inhalation of PC945 5 mg in healthy subjects (Cohort 1) and subjects with mild asthma (Cohort 3)

Following single inhaled administration of PC945 at 5 mg, a geometric mean *C*_max_ of 335 pg/mL in plasma was achieved 4 hours (median *t*_max_) postdose (Figure 4). Between‐subject variability in the extent of systemic exposure to PC945 was moderate to high, with geometric CVs for *C*_max_ and AUC_0–24h_ of 68.7% and 49.7%, respectively. Systemic exposure to PC945 (*C*_max_ and AUC_0–24h_) in subjects with mild asthma (335 pg/mL and 4950 pg*h/mL, respectively) was not meaningfully different from that in healthy subjects at 5 mg (322 pg/mL and 5440 pg*h/mL, respectively).

##### Repeat dose in healthy subjects (Cohort 2)

Following a single inhaled administration of PC945 5 mg on Day 1, a geometric mean *C*_max_ of 462 pg/mL in plasma was achieved 2 hours (median *t*_max_) postend of inhalation (Table 10 and Figure 3).

**TABLE 10:** Geometric mean (CV%) pharmacokinetic parameters of PC945 following repeat doses of PC945 5 mg in healthy subjects (Cohort 2)

PK Parameter	Day 1	Day 7
*C*_max_ (pg/mL)	462 (68.0)	951 (58.7)
*t*_max_ (h)	2.10 (2.08‐4.08)	0.745 (0.240‐10.1)
AUC_0–24h_ (pg*h/mL)	6510 (60.0)	17 200 (60.9)
*t*_½_ (h)	–	132 (22.7)
R_O_		2.64 (86.0)

Note

For *t*_max_, data are presented as median (range).

Abbreviations: AUC_0–24h_, area under the plasma concentration‐time curve from time zero to 24 hours; *C*_max_, maximum plasma concentration; CV, coefficient of variation; R_O_, accumulation ratio; *t*_½_, apparent terminal half‐life; *t*_max_, time at which *C*_max_ occurs.

**FIGURE 5:**
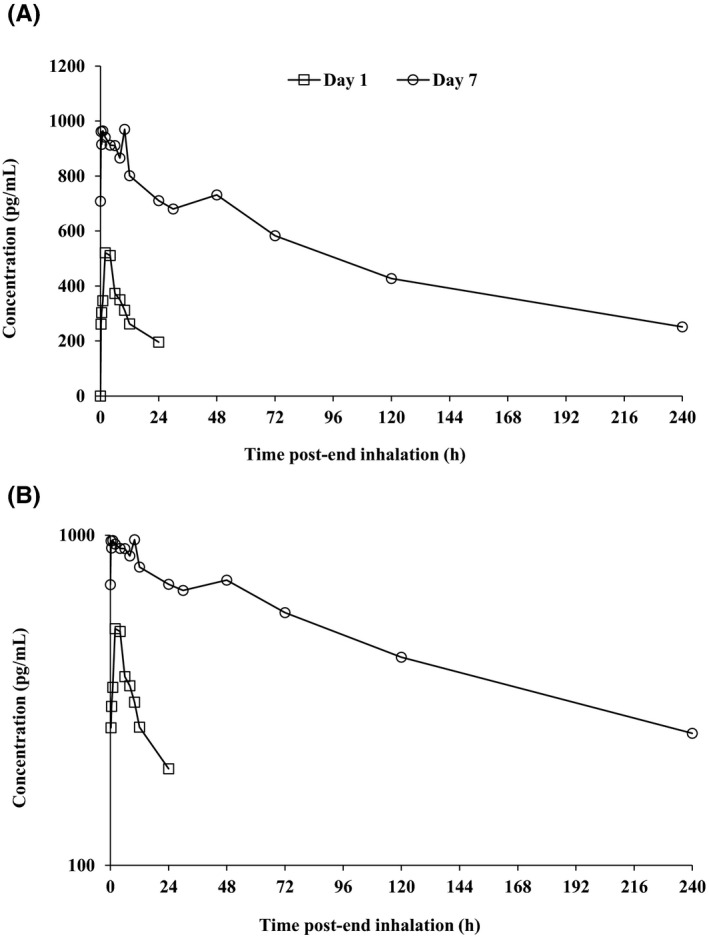
Mean plasma concentrations (linear [A] and log scales [B]) vs time profiles of PC945 following repeat daily administration of PC945 5 mg in healthy subjects (Cohort 2, Day 1 and Day 7)

Following repeat daily inhaled administration of PC945 5 mg, a geometric mean *C*_max_ of 951 pg/mL was achieved at 45 minutes postend of inhalation on Day 7. Systemic exposure to PC945 (AUC_0–24h_) increased 2.6‐fold following 7‐day dosing (Figure 5); visual inspection of predose concentrations indicated that steady state had not been reached by Day 7 (data not presented). Between‐subject variability in the extent of systemic drug exposure was moderate with geometric CVs for *C*_max_ and AUC_0–24h_ ranging from 58.7% to 60.9%.

##### Dose proportionality

Dose proportionality was only assessed for single doses using *C*_max_ and AUC_0–24h_. Systemic exposure to PC945 increased with escalating doses from 0.5 to 10 mg in an approximately dose‐proportional manner. For a doubling in dose, *C*_max_ and AUC_0–24h_ values increased 1.77‐fold and 1.88‐fold; the 90% confidence interval was within the prescribed limits of 1.6 to 2.5 for AUC_0–24h_ (values 1.64, 2.15) indicating dose proportionality, though just outside the lower limit for *C*_max_ (values 1.54, 2.02).

## 4 DISCUSSION

PC945 is a novel antifungal triazole, designed specifically for inhaled administration with physico‐chemical properties for sustained lung retention and persistent antifungal activity.^13^ Drug delivery directly to the lung to treat pulmonary disease is well established.^22^, ^23^ This dosing route maximizes local efficacy in the lungs whilst minimizing the potential for adverse systemic effects or drug interactions. Administration of a drug solution into the lung leads to rapid absorption into the systemic circulation; consequently strategies for developing effective inhaled medicines have focused on lipophilic compounds that have low aqueous solubility and slow dissolution rates in aqueous media.^24^ Such compounds are typically delivered to the lungs in a micronized, crystalline form as either an aqueous suspension, a pressurized metered dose inhaler or a dry powder blended with a carrier such as lactose. The slow dissolution of inhaled drug particles enhances local action by prolonging lung retention, whilst simultaneously delaying the rate of delivery to the systemic circulation. This strategy was adopted in the development of PC945.^13^

Following single inhaled doses in rats (Day 1 data), PC945 showed a slow and sustained period of absorption into the systemic circulation such that *C*_max_ occurred 6 hours after the start of dosing. Similarly, in dogs, there was a slow rate of PC945 absorption from the lung followed by sustained plasma levels that extended beyond 24 hours after the initial dose. Systemic exposure was approximately dose‐proportional in both test species. The slow rate of PC945 absorption indicated that accumulation of PC945 would occur with repeat daily dosing in rats and dogs. Repeat dose studies confirmed that dose absorption from the lungs was a rate‐limiting process in the pharmacokinetic profile of inhaled PC945.

The pharmacokinetic profile of PC945 in humans showed similar drug behavior to that observed in the nonclinical species investigated. Systemic exposure to PC945 in humans (*C*_max_ and AUC_0–24h_) increased with escalating doses in a dose‐proportional manner and following 7‐day, once daily dosing with 5 mg PC945 and accumulation was observed. Visual inspection of predose plasma concentrations indicated that steady state had not been reached by Day 7, and was predicted to be attained approximately 5 weeks after once daily dosing. The observed prolonged plasma *t*_½_ was consistent with slow absorption from the lung, demonstrating that a typical lung‐dominant process was controlling systemic kinetic behavior. The t_max_ data across the cohorts suggested rapid exposure to the respiratory epithelium following the inhaled delivery.

In rat lung samples on Day 15 after 14‐day inhaled dosing, mean concentrations of PC945 were approximately proportional to daily doses and >2000‐fold (range 2340‐5020) higher than the concentrations in plasma. The pharmacokinetic data obtained in rats and dogs indicate that the rates of dissolution and subsequent absorption of PC945 from the lungs are the principal factors determining systemic exposure to PC945 following inhaled dosing. Based on the mean lung:plasma ratio observed in male rats (~4300 fold) lung concentrations of PC945 in humans following a 5 mg single dose were estimated to be in the region of the 90% of the minimal inhibitory concentration for clinical strains of *A fumigatus* (1 µg/mL).^13^

One potential factor affecting distribution and clearance of PC945 in the lungs is uptake into macrophages in the alveolar space and/or epithelial cells. Several studies in humans investigating the intrapulmonary pharmacokinetics of azole antifungal agents, such as itraconazole, voriconazole and posaconazole, have been reported.^25^, ^26^, ^27^, ^28^ For itraconazole and posaconazole, preferential distribution into alveolar cells could be a favorable factor for successful treatment and prevention of respiratory fungal disease. For example, 14‐day oral administration of posaconazole (400 mg twice daily) resulted in steady state in which mean concentrations were over 30‐fold greater in pulmonary alveolar cells than in lung epithelial lining fluid and plasma, with an alveolar cell:plasma ratio of 27 to 44 after the last dose.^27^ Similar pulmonary pharmacokinetic data have been reported with oral posaconazole treatment in lung transplant patients, suggesting that uptake into alveolar cells is a reproducible distribution characteristic of the compound in clinical studies.^28^ Preferential uptake of antifungal agents into alveolar cells (which are principally macrophages) could be clinically relevant, as macrophages are a host defence mechanism against alveolar infection by *Aspergillus* and scavenge to remove particulate matter.^7^, ^29^

It is well known that all antifungal azoles are potent inhibitors of CYP450 enzymes, leading to a significant risk of interactions with other comedicated drugs.^7^, ^30^ All of these agents potently inhibit human CYP3A4 isoforms and the inhibitory activity is extended to human CYP2C9 and CYP2C19 in the cases of fluconazole and voriconazole.^7^ It is also reported that posaconazole has the potential to inhibit CYP450‐mediated steroid hormone synthesis causing hypertrophy or hyperplasia of the adrenal glands.^31^ These CYP inhibitory actions are well established as a mechanism of pharmacokinetics‐based drug‐drug interactions in the clinic.^7^ PC945 is a potent inhibitor of human CYP3A4/5. However, oral inhalation of PC945 resulted in low systemic exposure as demonstrated by a mean plasma *C*_max_ of 951 pg/mL [equivalent to 0.0016 µmol/L] following 7‐day, once daily, 5 mg doses, an order of magnitude lower than the inhibitory IC_50_ value (0.017 µmol/L; determined as the time‐dependent value for in vitro inhibition of midazolam in pooled human liver microsomes). Moreover, the *C*_max_ determined for PC945 [0.0016 µmol/L] is much lower than that of either itraconazole (1.52 µmol/L after 15‐day, 200 mg daily oral dosing) or posaconazole (0.83 µmol/L after 200 mg three times daily oral dosing).^32^, ^33^ The low *C*_max_ values of <0.002 µmol/L obtained with inhaled doses of PC945 suggest it will have a much lower drug‐drug interaction risk than that of orally administered triazole antifungals.

In the first‐in‐human study, reported herein, PC945 was well tolerated in healthy subjects at either single doses up to 10 mg or at repeat doses of 5 mg once daily for 7 days in addition to subjects with mild asthma at a single dose of 5 mg. No evidence of irritancy was observed in subjects with mild asthma.

In summary, PC945 was well tolerated at all doses tested and for a duration up to 7 days; no safety signals were identified. The pharmacokinetic profile translated well from nonclinical species to results derived from the clinic. Key findings of the study reveal that PC945 undergoes slow absorption from the lung and exhibits low systemic exposure thus limiting the potential for an adverse side effect profile and drug interactions commonly seen with oral azoles. In addition, systemic exposure to PC945 in subjects with mild asthma showed no appreciable difference in its profile to that observed in healthy subjects at the same dose level, suggesting no dose adjustment would be anticipated in subsequent studies in patients. We demonstrated that 5 mg is a suitable dose and the data, together with a case report describing the treatment of fungal tracheobronchitis post lung transplantation, support the further development of PC945 in studies of patients with pulmonary aspergillosis.^34^

## DISCLOSURES

All authors have completed the Unified Competing Interest form at http://www.icmje.org/coi_disclosure.pdf (available on request from the corresponding author). Pulmocide Ltd. provided funding for this clinical trial. LC, AM and AD are employees of Pulmocide Ltd and own stock options of Pulmocide Ltd. KI and PS are (co)founders and employees of Pulmocide Ltd, and own stock options of Pulmocide Ltd. JM and GR are (co)founders and consultants of Pulmocide Ltd. and own stocks of Pulmocide Ltd. KW, JA, JP, EF, PH, and CW are part‐time consultants to Pulmocide Ltd.

## AUTHOR CONTRIBUTIONS

LC, AM, GR, PS, JP, JA, AD, EF, and PH contributed to the design of the studies and the protocols. MA conducted the study. KW, AD, and LC directed the studies at Pulmocide. AM, CB, and JA analyzed/interpreted the data, JM, CW, and KI critically revised the manuscript, agreed on the content, and approved the final version of publication.

## Data Availability

The data that support the findings of this study are available from the corresponding author upon reasonable request.
